# The role of surgical intervention in primary colorectal lymphoma: A SEER population-based analysis

**DOI:** 10.18632/oncotarget.12344

**Published:** 2016-09-29

**Authors:** Yi-bo Cai, Hai-yan Chen, Jin-jie He, Ye-ting Hu, Qi Yang, Liu-bo Chen, Qian Xiao, Ke-feng Ding

**Affiliations:** ^1^ Department of Surgical Oncology, The Second Affiliated Hospital, Zhejiang University School of Medicine, Hangzhou, Zhejiang, China; ^2^ Cancer Institute, Key Laboratory of Cancer Prevention and Intervention, China National Ministry of Education, Key Laboratory of Molecular Biology in Medical Sciences, The Second Affiliated Hospital, Zhejiang University School of Medicine, Hangzhou, Zhejiang, China

**Keywords:** primary colorectal lymphoma, SEER, prognosis, surgical intervention, survival

## Abstract

**Background:**

Primary colorectal lymphoma (PCL) is a rare colorectal malignancy. The standard treatment and prognostic factors of PCL remain unexplored. Therefore, a large population-based study should be conducted to provide a detailed review of this disease.

**Methods:**

We extracted the data of eligible patients with PCL registered in the SEER database from 1973 to 2011. All statistical analyses were performed using SPSS 19.0.

**Results:**

A total of 2050 (61.3%) of the 3342 patients with PCL underwent surgical intervention, and 1292 (38.7%) patients received no surgical treatment. The median overall survival was 95 months, and patients receiving surgery exhibited significantly prolonged survival (adjusted HR =0.69, *P* <0.001). Young age, early tumor stage, and indolent lymphoma were independent predictors of improved survival. Further survival analyses demonstrated the potential benefit of surgery in patients with early tumor stage, right-sided lesions, or diffuse large B-cell PCL. Conversely, surgical intervention did not improve the survival of patients with advanced-stage, left-sided, or indolent PCL.

**Conclusion:**

PCL is a rare tumor that can be effectively treated. Surgical intervention may play an important role in the treatment of PCL. Early tumor stage, a right-sided lesion, and diffuse large B-cell histological PCL seem to be the clinical characteristics of optimal surgical candidates.

## INTRODUCTION

Involvement of extranodal organs is a relatively common clinical finding in Non-Hodgkin lymphoma (NHL), and approximately 25-40% of patients present with primary extranodal lymphoma [1]. The majority of extranodal involvement, which accounts for 30-40%, occurs in the gastrointestinal tract [2–4]. Primary colorectal lymphoma (PCL) is an uncommon original site of extranodal NHL that accounts for approximately 10-20% of gastrointestinal NHL [5–7] and comprises 0.2-0.6% of large bowel malignancies [8]. The annual incidence of colonic lymphoma has increased more than two-fold from 1.0 per 1 million in 1973 to 2.3 per 1 million in 2004 [9]. However, the diagnostic criteria of PCL are not standardized because of the rarity of PCL and lack of clinical data.

Clinical manifestations of PCL seem to be closely correlated with tumor location [10]. Abdominal pain is perceived as the most common symptom. Changes in bowel habits and bloody stools are also frequently encountered [11, 12]. It is difficult to differentiate PCL from typical digestive diseases in adults because of these non-specific clinical symptoms. PCL is often not discovered until serious complications arise, such as perforation or obstruction [10]. Cai et al. [13] reported that more than half of the patients with PCL underwent emergency surgeries for serious complications. Therefore, surgeons should remain vigilant about the diagnostic possibility of PCL in emergent patients. Inflammatory bowel disease and immunosuppression are considered potential risk factors of PCL [14, 15]. The optimal therapeutic approach for the treatment of PCL remains controversial because PCL is rarely encountered in clinical practice, and few random controlled trials have been performed. Historically, patients with PCL were managed using a multidisciplinary approach with surgery, chemotherapy and radiotherapy in selected patients [13, 15, 16]. However, there is no enough evidence to support a correlation of surgical intervention with better long-term survival, and the relevant factors affecting surgical clinical efficacy in the treatment of PCL remain unexplored. Information on the epidemiology, surgical treatment and prognosis of PCL is limited because of the rarity of this disease. Therefore, the main purpose of this retrospective study based on the authoritative Surveillance, Epidemiology, and End Results (SEER) database is to provide the best available information to improve clinicians' understanding of PCL. This study also revealed the potential predictive factors of surgical outcomes and evaluated the role of surgery to a certain degree.

## RESULTS

### Patient demographics and tumor characteristics

**Figure 1:**
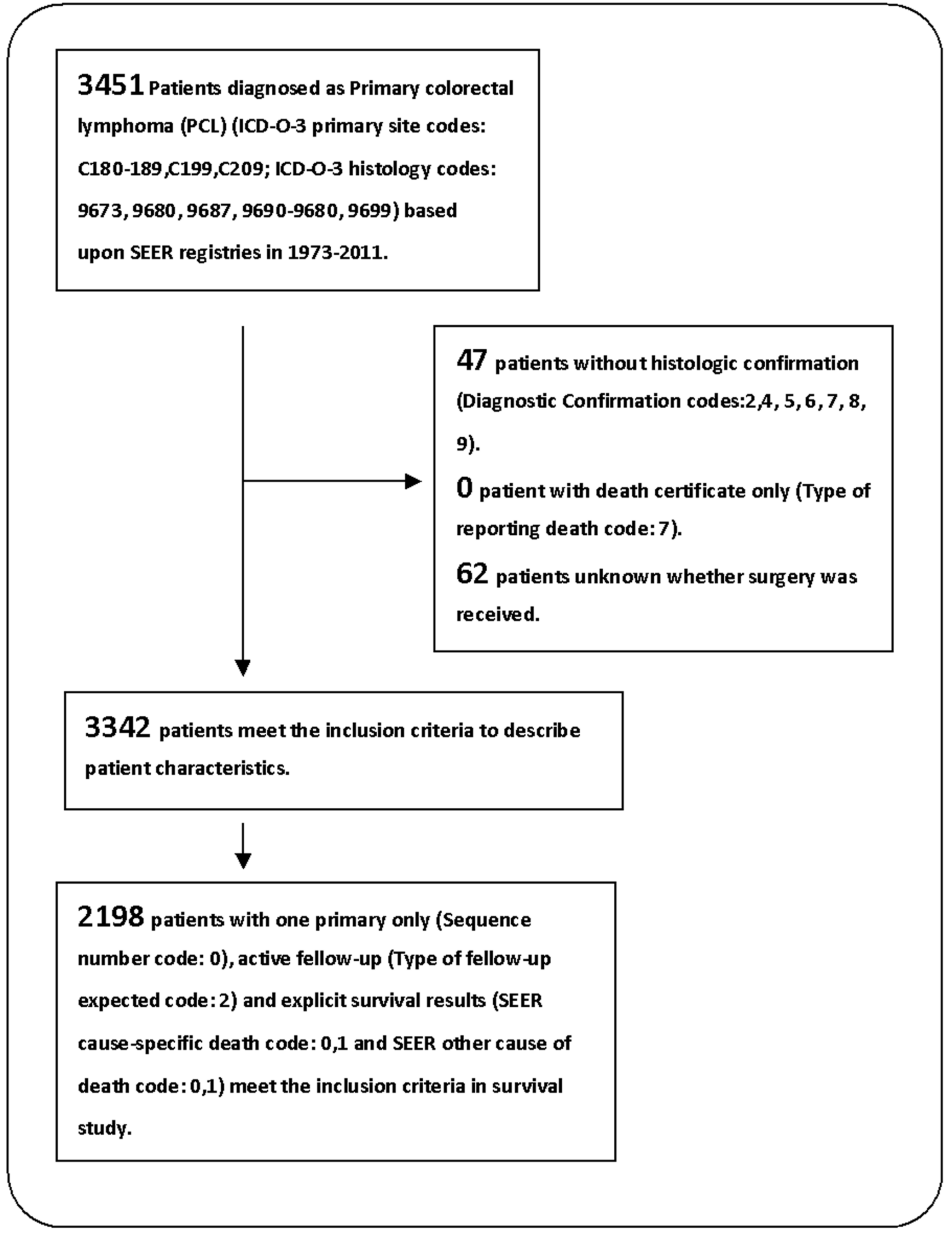
Flow diagram of patient inclusion and exclusion 3342 patients with primary colorectal lymphoma were identified based upon SEER database from 1973 to 2011, and 2198 of them were further extracted for survival analysis.

A total of 4525 patients with PCL were identified in the SEER database from 1973 to 2011. Considering the heterogeneous nature of lymphoma, we focused on the five most common histological subtypes Table 1), which accounted for 76.3% of all patients with PCL in the SEER database. Of the candidate patients, 3342 cases met the inclusion criteria (Figure 1), including 2050 cases (61.3%) with surgical intervention and 1292 cases (38.7%) without surgical intervention. The demographics, tumor characteristics, and treatment in formation for these two groups were summarized in Table 2. The mean age of patients at PCL diagnosis was 63.9 years old (SD = 18.3 years old). Male patients comprised a larger proportion (*N* = 2046, 61.2%), and most patients were white (*N* = 2275, 83.0%). Approximately 41.8% of PCL patients were categorized as stage IE (*N* = 1396), 23% as stage IIE (*N* = 767), 5% as stage IIIE (*N* = 168), and 21.3% as stage IVE (*N* = 712). Most patients who underwent surgical intervention were diagnosed at stage IIE (*P* < 0.001), whereas fewer patients were in stage IVE (*P* < 0.001). A total of 1165 cases (34.9%) originated from the cecum, which comprised the largest proportion. The two most common lymphoma types were diffuse large B-cell (*N* = 1828, 54.7%) and marginal zone B-cell (*N* = 618, 18.5%). Approximately 56.6% of PCL patients (*N* = 1890) underwent surgical intervention. The rate of surgery presented a descending trend from 94.2% (257/274) in the period 1973-1990 to 54.9% (735/1324) in the period 2006-2011. Radiotherapy was only performed in 4.5% (*N* = 152) of patients with PCL.

**Table 1:** The distribution of histologic types in PCL

ICD-O-3 Code	Histologic type	Number	Percent
9590	Malignant lymphoma, NOS	213	4.7
9591	Malignant lymphoma, non-Hodgkin	328	7.2
9596	Composite Hodgkin and non-Hodgkin lymphoma	1	0.0
9650	Hodgkin lymphoma, NOS	18	0.4
9652	Hodgkin lymphoma, mixed cellularity, NOS	9	0.2
9653	Hodgkin lymphoma, lymphocytic deplet., NOS	3	0.1
9659	Hodgkin lymph., nodular lymphocyte predom.	1	0.0
9663	Hodgkin lymphoma, nodular sclerosis, NOS	1	0.0
9670	ML, small B lymphocytic, NOS	118	2.6
9671	ML, lymphoplasmacytic	21	0.5
**9673**	**Mantle cell lymphoma**	**329**	**7.3**
9675	ML, mixed sm. and lg. cell, diffuse	41	0.9
**9680**	**ML, large B-cell, diffuse**	**1886**	**41.7**
9684	ML, large B-cell, diffuse, immunoblastic, NOS	182	4.0
**9687**	**Burkitt lymphoma, NOS**	**243**	**5.4**
**9690**	**Follicular lymphoma, NOS**	**120**	**2.6**
**9691**	**Follicular lymphoma, grade 2**	**67**	**1.5**
**9695**	**Follicular lymphoma, grade 1**	**125**	**2.8**
**9698**	**Follicular lymphoma, grade 3**	**42**	**0.9**
**9699**	**Marginal zone B-cell lymphoma, NOS**	**639**	**14.1**
9702	Mature T-cell lymphoma, NOS	37	0.8
9714	Anaplastic large cell lymphoma, T-cell and Null cell type	27	0.6
9717	Intestinal T-cell lymphoma	8	0.2
9719	NK/T-cell lymphoma, nasal and nasal-type	4	0.1
9724	SystemicEBV pos. T-cell lymphoproliferative disease of childhood	1	0.0
9727	Precursor cell lymphoblastic lymphoma, NOS	3	0.1
9731	Plasmacytoma, NOS	19	0.4
9734	Plasmacytoma, extramedullary	36	0.8
9735	Plasmablastic lymphoma	11	0.2
Total patients with PCL listed in SEER (1973–2011)		4525	100.0
**Patients selected for analysis** ^a^		**3451**	**76.3**

Abbreviation: PCL, Primary Colorectal Lymphoma; ICD-O-3, International Classification of Diseases for Oncology, 3rd Edition; NOS, Not Otherwise Specified; ML, Malignant lymphoma; SEER, Surveillance, Epidemiology, and End Results.

a ICD-O-3 Histological Type Codes of selected patients in this analysis are 9673, 9680, 9687, 9690, 9691, 9695, 9698, and 9699, which are marked in bold.

**Table 2:** Characteristics of patients with PCL

Variable	All Patients	Surgery	No Surgery	*P* Value
Total Patients (%)	3342(100%)	2050(61.3%)	1292(38.7%)	
				
Age (SD)	63.9±18.3	63.4±18.9	64.5±17.2	0.501 ^a^
Gender (%)				0.772 ^b^
Male	2046(61.2%)	1259(61.4%)	787(60.9%)	
Female	1296(38.8%)	791(38.6%)	505 (39.1%)	
Race (%)				0.370 ^b^
White	2275(83.0%)	1714(83.6%)	1061(82.1%)	
Black	189(5.7%)	108(5.3%)	81(6.3%)	
Asian	320(9.6%)	197(9.6%)	123(9.5%)	
Others	58(1.7%)	31(1.5%)	27(2.1%)	
Stage (%)				<0.001 ^b^
Stage IE	1396(41.8%)	826(40.3%)	570(44.1%)	0.029 ^b^
Stage IIE	767(23.0%)	597(29.1%)	170(13.2%)	<0.001 ^b^
Stage IIIE	168(5.0%)	88(4.3%)	80(6.2%)	0.014 ^b^
Stage IVE	712(21.3%)	379(18.5%)	333(25.8%)	<0.001 ^b^
Not applicable	114(3.4%)	109(5.3%)	5(0.4%)	<0.001 ^b^
Unknown	185(5.5%)	51(2.5%)	134(10.4%)	<0.001 ^b^
Radiation (%)				<0.001 ^b^
Yes	295(8.8%)	143(7.0%)	152(11.8%)	<0.001 ^b^
No	3025(90.5%)	1890(92.2%)	1135(87.8%)	<0.001 ^b^
Unknown	22(0.7%)	17(0.8%)	5(0.4%)	
Location (%)				<0.001 ^b^
Cecum	1165(34.9%)	797(38.9%)	368(28.5%)	<0.001 ^b^
Appendix	109(3.3%)	91(4.4%)	18(1.4%)	<0.001 ^b^
Right colon	386(11.5%)	247(12.0%)	139(10.7%)	
Hepatic flexure	65(1.9%)	43(1.9%)	22(1.7%)	
Transverse colon	165(4.9%)	103(5.0%)	62(4.8%)	
Splenic flexure	41(1.2%)	32(1.6%)	9(0.7%)	0.027 ^b^
Left colon	91(2.7%)	58(2.8%)	33(2.6%)	
Sigmoid colon	320(9.6%)	188(8.9%)	132(10.2%)	
Overlappping lesion	100(3.0%)	62(3.0%)	38(2.9%)	
Colon, NOS	358(10.7%)	166(8.1%)	192(14.9%)	<0.001 ^b^
Rectosigmoid	96(2.9%)	47(2.3%)	49(3.8%)	0.011 ^b^
Rectum	446(13.3%)	216(10.5%)	230(17.8%)	<0.001 ^b^
Histology (%)				<0.001 ^b^
NHL, large B-cell	1828(54.7%)	1186(57.9%)	642(49.7%)	<0.001 ^b^
Marginal zone B-cell	618(18.5%)	358(17.5%)	265(20.5%)	0.028 ^b^
Burkitt lymphoma	233(7.0%)	154(7.5%)	79(6.1%)	
Follicular lymphoma	346(10.4%)	200(9.8%)	146(11.3%)	
Mantle cell lymphoma	317(9.5%)	157(7.7%)	160(13.4%)	<0.001 ^b^
Year of diagnosis (%)				<0.001 ^b^
1973-1990	274(8.2%)	257(12.5%)	17(1.3%)	<0.001 ^b^
1991-2000	753(22.5%)	478(23.3%)	275(21.3%)	
2001-2005	991(29.7%)	580(28.3%)	411(31.8%)	0.030 ^b^
2006-2011	1324(39.6%)	735(35.8%)	589(45.6%)	<0.001 ^b^

Abbreviation: PCL, Primary Colorectal Lymphoma; SD, Standard Deviation; NOS, Not Otherwise Specified; NHL, Non-Hodgkin's Lymphoma.

a Wilcoxon rank sum test.

b Pearson Chi-square test.

### Survival and prognostic factors

**Figure 2:**
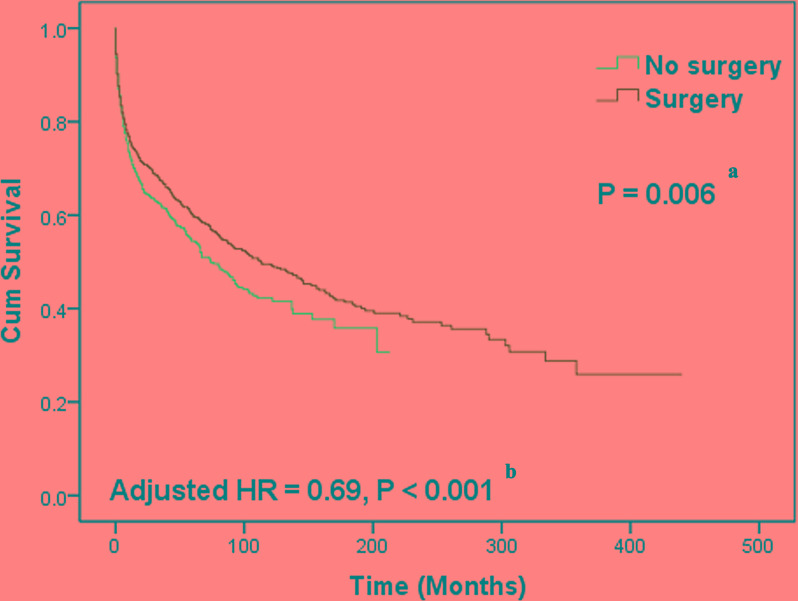
Kaplan-Meier curves of overall survival differences between patients with and without surgical intervention Patients undergoing surgical intervention could have prolonged survival. ^a^ Log-rank test was performed in univariate analysis to detect survival differences. ^b^ The Cox multivariate proportional hazard model was used to estimate adjusted hazard ratios (HRs).

Approximately 2198 cases (65.8%) with complete survival information were eligible for inclusion in accurate analyses of the overall survival (OS) of patients with PCL (Figure 1). The eligible study population was not significantly different from the total population (Table S1). The median OS was 95 months (range = 79.5-110.5 months) (Table 3). Younger patients exhibited improved prognosis compared to elderly patients (age ≤50 years versus >70 years, *P* < 0.001). Non-white patients exhibited prolonged survival (*P* = 0.027). Tumor stage was a crucial predictor of survival in PCL patients. Advanced tumor stage correlated with decreased survival (*P* < 0.001). Patients undergoing surgical intervention showed improved survival (113 months versus 74 months, *P* = 0.006) (Figure 2). Marginal zone B-cell and follicular histological types (*P* < 0.001), as well as year of diagnosis >2000 (*P* < 0.001), were potential predictors of better prognosis. Adjusted Cox regression analyses revealed that advanced stage and tumor localization in the left-sided colon were independent factors of poor prognosis (Table 3). Younger age, surgical intervention (adjusted hazard ratio (HR) = 0.69, 95% confidence intervals (CI): 0.59-0.81, *P* < 0.001), marginal zone B-cell lymphoma, follicular lymphoma, and year of diagnosis >2000 were independent predictors of improved outcomes. The local excision (LE) group (*N* = 617, 29.4%) was positively associated with survival [144 months versus 102 months for the LE and radical excision (RE) groups, respectively, *P* < 0.001] (Table S2). However, this survival difference was not confirmed in multivariate analysis (adjusted HR = 1.01, 95% CI: 0.83-1.21). Further analysis revealed that the number of patients with survival times shorter than 3 months in the LE group was lower than in the RE and no-surgery groups (*P* = 0.010). Most patients with stage IE PCL underwent LE (54.7%), while stage IVE PCL was the most common stage in patients without surgery (*P* < 0.001).

**Table 3:** Univariate and Multivariate Analyses for Overall Survival

Variable	Univariate		Unadjusted model		Adjusted model	
	Median survival] (95%CI, months)	P value^a^	HR	P value^b^	HR	P value^b^
**Overall**	95(79.5-110.5)					
**Age**						
≤ 50	221^c^		0.46(0.38-0.57)	<0.001	0.46(0.38-0.56)	<0.001
51-70	152(116.1-187.9)		0.54(0.46-0.64)	<0.001	0.56(0.48-0.66)	<0.001
>70	53(43.6-62.4)	<0.001	1		1	
**Gender**						
Male	103(82.7-123.3)		NA		NA	
Female	92(70.3-113.7)	0.527				
**Race**						
White	91(75.6-106.4)		1		NA	
Nonwhite	171(124.6-217.4)	0.027	0.95(0.78-1.16)	0.615		
**Stage**						
Stage IE	158(127.3-188.7)		1		1	
Stage IIE	92(60.6-123.4)		1.35(1.12-1.62)	0.002	1.34(1.12-1.61)	0.001
Stage IIIE	65(33.7-96.3)		1.69(1.24-2.30)	<0.001	1.64(1.21-2.23)	0.002
Stage IVE	35(18.7-51.3)	<0.001	2.13(1.79-2.53)	<0.001	2.13(1.79-2.52)	<0.001
**Radiation**						
No	99(82.0-116.0)		NA		NA	
Yes	88(62.1-113.9)	0.930				
**Surgery**						
No	74(59.8-88.2)		1		1	
Yes	113(91.1-134.9)	0.006	0.69(0.59-0.81)	<0.001	0.69(0.59-0.81)	<0.001
**Location**						
Right-sided colon	99(79.2-118.8)		1.16(0.94-1.43)	0.173	1.15(0.94-1.42)	0.180
Left-sided colon	80(53.2-106.8)		1.39(1.09-1.78)	0.008	1.37(1.07-1.74)	0.012
Rectum	121(73.3-168.7)	0.075	1		1	
**Histology**						
NHL, large B-cell	73(59.5-86.5)		1		1	
Marginal zone B-cell	170^c^		0.63(0.50-0.80)	<0.001	0.62(0.49-0.78)	<0.001
Burkitt lymphoma	221^c^		0.98(0.74-1.31)	0.904	1.00(0.75-1.33)	0.997
Follicular lymphoma	166(112.3-219.7)		0.66(0.50-0.88)	0.004	0.66(0.50-0.87)	0.003
Mantle cell lymphoma	70(37.5-102.5)	<0.001	0.87(0.66-1.14)	0.317	0.86(0.66-1.13)	0.279
**Year of diagnosis**						
1973-1990	38(15.3-50.7)		1		1	
1991-2000	50(32.9-67.1)		0.87(0.68-1.11)	0.268	0.88(0.69-1.12)	0.312
2001-2005	138^c^		0.52(0.40-0.68)	<0.001	0.53(0.41-0.69)	<0.001
2006-2011	153(107.7-198.3)	<0.001	0.53(0.41-0.69)	<0.001	0.54(0.41-0.69)	<0.001
Harrell concordance index^d^						0.681

Abbreviation: CI, Confidence Interval; NA, Not Applicable; NHL, Non-Hodgkin's Lymphoma.

a Median survival was determined by the Kaplan–Meier method. P value was calculated by Log Rank test.

b Hazard ratio and P value were determined by the Cox regression.

c Insufficient number of events to calculate the standard error of median overall survival.

d Harrell concordance index was performed to evaluate the goodness-of-fit of the finnal Cox model.

### Correlated factors associated with the effects of surgical intervention

**Figure 3:**
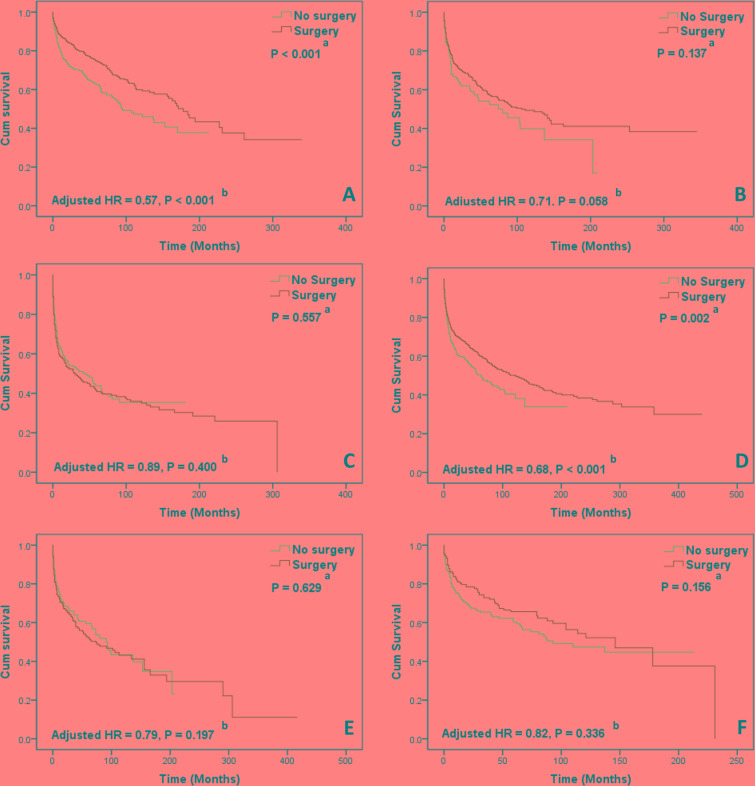
Kaplan-Meier curves of overall survival in subgroup analyses **A.** Patients with stage IE tumor, **D.** Patients with right-sided colonic lymphoma could benefit from surgical intervention and **B.** Patients with stage IIE tumor, **C.** Patients with stage IVE tumor, **E.** Patients with left-sided colonic lymphoma, **F.** Patients with rectal lymphoma did not achieve survival benefit from surgical intervention. ^a^ Log-rank test was performed in univariate analysis to detect survival differences. ^b^ The Cox multivariate proportional hazard model was used to estimate adjusted hazard ratios (HRs).

A subgroup analysis of survival was performed to determine the factors that correlated with the effect of surgical intervention, and the results are displayed in Table 4. Age had no effect on surgical efficacy in PCL. Patients in the stage IE PCL (adjusted HR = 0.57, 95% CI: 0.44-0.73) subgroup who underwent surgery exhibited an improved survival rate by several years, but this survival benefit from surgery was lost in stages IIE-IVE PCL. The surgery group showed improved survival in patients with tumors localized in the right-sided colon (adjusted HR = 0.68, 95% CI: 0.53-0.80) compared to the no-surgery group. The correlation between surgery and histological type was complicated. The survival benefit of surgery was only observed in the diffuse large B-cell PCL subtype (adjusted HR = 0.65, 95% CI: 0.54-0.82). These results are illustrated in the Kaplan-Meier survival curves in Figure 3. No significant survival improvement was found in the remaining cohorts (left-sided colon, rectum, marginal zone B-cell lymphoma, follicular lymphoma, Burkitt lymphoma, and mantle cell lymphoma) with surgical intervention.

**Table 4:** Analysis of Effects of Surgical Treatment on Overall Survival

Variable	Surgery		No Surgery		P ^a^	Hazard ratio	P ^b^
	Number(%)	10-year OS	Number(%)	10-year OS			
**Overall**	1360(61.9)	0.495	838(38.1)	0.422	0.006	0.69(0.59-0.81)	<0.001
**Age**							
≤50	342(63.9)	0.649	193(36.1)	0.473	0.001	0.66(0.47-0.93)	0.018
51-70	508(60.7)	0.552	329(39.3)	0.503	0,122	0.68(0.51-0.91)	0.010
>70	510(61.7)	0.317	316(38.3)	0.300	0.951	0.74(0.59-0.94)	0.011
**Stage**							
Stage I E	531(58.9)	0.599	371(41.1)	0.473	<0.001	0.57(0.44-0.73)	<0.001
Stage II E	405(80.2)	0.493	100(19.8)	0.399	0.137	0.71(0.50-1.01)	0.058
Stage III E	62(52.1)	0.518	57(47.9)	0.353	0.138	0.80(0.43-1.50)	0.485
Stage IV E	258(53.4)	0.359	225(46.6)	0.353	0.557	0.89(0.67-1.17)	0.400
**Location**							
Right-side colon	870(68.4)	0.501	402(31.6)	0.405	0.002	0.68(0.53-0.80)	<0.001
Left-side colon	214(61.3)	0.432	135(38.7)	0.434	0.629	0.79(0.56-1.13)	0.197
Rectum	176(49.4)	0.544	180(50.6)	0.474	0.156	0.82(0.55-1.22)	0.336
**Histology**							
NHL, large B-cell	787(64.8)	0.434	428(35.2)	0.381	0.019	0.65(0.54-0.82)	<0.001
Marginal zone B-cell	216(57.9)	0.624	157(42.1)	0.463	0.109	0.86(0.55-1.36)	0.523
Burkitt lymphoma	130(67.7)	0.652	62(22.3)	0.438	0.031	0.86(0.48-1.53)	0.599
Follicular lymphoma	128(58.7)	0.562	90(41.3)	0.590	0.959	0.72(0.39-1.32)	0.288
Mantle cell lymphoma	99(49.5)	0.454	101(50.5)	0.375	0.573	0.95(0.56-1.61)	0.843
**Year of diagnosis**							
1973-1990	194(95.1)	0.340	10(4.9)	0.000	0.033	1.41(0.49-4.08)	0.529
1991-2000	305(62.8)	0.402	181(37.2)	0.300	0.031	0.69(0.52-0.92)	0.011
2001-2005	372(58.4)	0.609	265(41.6)	0.479	0.008	0.73(0.54-0.98)	0.034
2006-2011	489(56.1)	0.575	382(43.9)	0.467	0.015	0.70(0.54-0.92)	0.009

Abbreviation: CI, Confidence Interval; OS, Overall Survival; NHL, Non-Hodgkin's Lymphoma.

a 10-year overall survival rate was determined by the Kaplan–Meier method. P value was calculated by the Log Rank test.

b Hazard ratio and P value was calculated by Cox proportional hazards model.

## DISCUSSION

PCL is a rare colorectal neoplasm with a gradually increasing incidence [9, 11, 17]. Previous studies that investigated the epidemiological features and survival of PCL were predominantly based on populations from a single institution and case reports; these studies only involved 7-95 patients [8, 10–13, 16, 18–26]. We identified 3342 patients with PCL in the SEER database to identify potential prognostic factors and the role of surgical intervention in PCL management. To our knowledge, this study is the largest population-based study to investigate factors associated with surgical efficacy for this relatively rare tumor.

Our results demonstrated that patients were diagnosed at the mean age of 63.9 years with a male predominance (male: female = 1.58: 1), which is similar to previous studies in the West and East [10, 12, 18, 26]. Approximately 41.8% and 21.3% of PCL patients were in Ann Arbor stages IE and IVE, respectively, which is similar to the results from a German multicenter trial [27]. Consistent with published reports [8, 10, 11, 16, 20], the most frequent tumor location was the cecum (34.9%), which primarily results from the abundance of lymphoid tissues in the lesion. The most common histological subtype in the present study was diffuse large B-cell (41.6%), and the proportion of T-cell lineage from the present results was lower than 17.9%, which was reported previously by the Korean Association for the Study of Intestinal Diseases [12]. Racial differences may partially explain this lack of conformity.

Age at diagnosis was an important predictor of survival, and younger patients (age ≤50 years) exhibited improved survival outcomes. This finding was corroborated by evidence from Droletet al. [11]. Early tumor stage and indolent lymphoma, which are good prognostic factors of survival in primary gastrointestinal lymphoma[7], were also associated with improved survival in PCL. This association between aggressive biological behavior and reduced survival was previously observed by Fan et al. [8], who demonstrated that patients with intermediate- or low- histological grade cancer showed prolonged survival compared to patients with high-grade cancer (132.0 months versus 62.2 months, P = 0.05). An increasing survival trend was observed over time, which may be due to improvements in diagnostic techniques and a multimodality approach to treatment. In particular, the rapidly developing targeted therapy for CD20-positive B lymphoma has further improved survival in lymphoma patients [28, 29].

Current PCL treatment has evolved into a combined therapy that involves surgery, chemotherapy, and radiotherapy [10, 13, 16, 23]. However, the role of surgery in this comprehensive PCL therapy strategy is still subject to debate. Most authors suggest surgery as the optimal initial treatment, regardless of clinical stage, because they believe that surgery provides important prognostic information, prevents complications, and provides the possibility of cure with or without adjuvant therapy [8, 11, 16, 24, 26]. Nevertheless, other investigators argue that NHL is a systemic disease and that surgical cure is unsuitable for advanced gastrointestinal lymphoma. Koniaris et al. [30] and Cai et al. [13] recommend systemic chemotherapy as the standard treatment for PCL and suggest that surgery should be reserved for PCL with serious complications, such as hemorrhage, obstruction and perforation. Nonetheless, because that the details of chemotherapy are unavailable from the SEER database, we are incapable to evaluate the actual role of chemotherapy in the treatment of PCL. The present results demonstrated that patients who received surgical intervention exhibited significant survival improvement (113 months versus 74 months, adjusted HR = 0.69, 95% CI: 0.59-0.81) and were consistent with previous study [26], supporting the potential important role of surgery in the comprehensive therapy of PCL.

Patients receiving surgical intervention should be assessed and selected according to their clinical and tumor features. A survival benefit from surgery was noted in patients with stage IE PCL in the present study, but this benefit was lost in patients with advanced tumor stage, which is consistent with the results reported in gastric lymphoma [31]. This finding was also corroborated by evidence from Cai et al. [13], who demonstrated that all patients with stage IVE PCL had disease progression after emergency surgery. Therefore, some oncologists recommend that patients with aggressive tumors in stages IIIE-IVE should be referred to clinical trials rather than undergoing surgery or chemotherapy [10, 15].

The location of extranodal NHL seems to be associated with surgical efficacy. Surgical intervention in colorectal lymphoma exerted a significant positive effect on survival outcomes of right-sided [right-sided colonic (adjusted HR = 0.68, *P* < 0.001)] PCL but lost efficacy in left-sided [left-sided colonic (adjusted HR = 0.79, *P* = 0.197) or rectal (adjusted HR = 0.82, *P P* = 0.336)] PCL. This result may be attributed to the following reasons. First, radical surgeries performed in right-sided and left-sided PCL are different and result in varied degrees of postoperative complications. A larger population-based study from the University HealthSystem Consortium Clinical Database revealed that right-sided colectomy led to fewer complications than left-sided colectomy for malignancy (26.8% versus 28.3%, *P* < 0.05) [32]. Kwaan et al. [33] also reported that a high rate of superficial surgical site infection was found in left-sided colectomy. Therefore, we hypothesize that an increased complication rate would counteract the positive efficacy of surgical intervention in left-sided PCL. Second, the different surgical effect by tumor site is likely related to the biological characteristics of the tumor. The embryological midgut develops into the right-sided colon during development (including from the cecum to the proximal two-thirds of the transverse colon), and the embryological hindgut develops into the left-sided colon (including from the distal third of the transverse colon to the upper anal canal) [34]. The biological characteristics and gene variations may differ between right-sided and left-sided PCL, which may affect the therapeutic response to chemotherapy or targeted therapy. Ferreri et al. [35] investigated the role of surgical intervention in the therapy of gastric lymphoma by reviewing retrospective and prospective studies and suggested that nonsurgical treatments, such as chemotherapy, achieved equal or better outcomes than gastrectomy. Hence, considering the larger injury and high risk of permanent stoma caused by left-sided colorectal radical surgery, we suggest that surgeons should circumspectly recommend radical surgery for left-sided PCL. Combined chemotherapy with or without target therapy may be as effective as surgical intervention in these patients.

Radical resection has been the primary method for surgery of PCL, but the efficacy of this technique has not been proven in any clinical trials or retrospective studies. The present data demonstrated that approximately 29.4% of PCL patients underwent LE, which contradicts previous reports. Notably, univariate analysis revealed significantly prolonged survival in patients who underwent LE (*P* < 0.001), but this survival benefit was lost in patients who received RE (*P* = 0.177). We hypothesize that increased postoperative mortality counteracts the positive long-term effect of radical resection. However, the fact that patients who received LE were diagnosed at an earlier stage and were slightly younger should not be ignored. This survival difference was likely disturbed by selection bias, which arose from stage and age migration. Our failure to confirm this result in multivariate analysis and the unavoidable limitations in the current study prevent us from drawing the conclusion that patients who underwent LE exhibit improved survival. LE has the advantages of fewer operative injuries, faster recovery, cost savings, and avoidance of permanent stoma compared to RE. Therefore, a large-scale prospective clinical trial must be performed to reevaluate the practical role of local and radical surgery in PCL treatment.

This study presents several limitations. First, important clinical information, such as chemotherapy data, surgical details, and treatment-related complications, were not released in the SEER standard data. Therefore, several potential prognostic factors were not considered in the analysis. Second, intrinsic limitations exist in the SEER database because of variations in data reporting and coding systems, patient migration, and selection bias [36]. The absence of centralized meticulous histopathology reports or reviews is another inextricable limitation of the SEER program. NHL encompasses a heterogeneous group of histological presentations, and the consistency and accuracy of pathology reports provided by multiple participating institutions are difficult to ensure. Therefore, we cannot ignore the potential misclassification of the patients included in this study.

## CONCLUSIONS

Using a large nationwide cancer database, this study revealed that age, tumor stage, and histological type maybe independent predictors of PCL prognosis. Surgical intervention was associated with survival. Surgical intervention did not improve survival in patients with an advanced tumor stage, left-sided lesion, or indolent PCL, but early tumor stage, right-sided lesion, or diffuse large B-cell histological type seems to be the clinical characteristics of optimal surgical candidates. However, a prospective study must be performed to confirm these findings. An ideal individualized therapy could maximize survival benefit in patients.

## MATERIALS AND METHODS

### Data source

The SEER program is supported by the National Cancer Institute and collects data on cancer incidence and survival from 18 participating population-based cancer registries. This program covers about 28% of the US population, which is approximately 86.4 million people, and updates information annually, including patient demographics, tumor characteristics, treatment and follow-up data [37]. The current study used the SEER database April 2014 release to identify all patients with PCL from 1973 to 2011. Information on patient demographics, tumor characteristics, surgery, radiation and survival data were extracted from this database. The site of the primary tumor was classified into right-sided colon (including cecum, appendix, right colon, hepatic flexure and transverse colon), left-sided colon (including splenic flexure, left colon and sigmoid colon) and rectum (overlapping lesions and rectum) in further survival study. Our analyses used the Ann Arbor staging from the SEER program as the staging criterion.

We partitioned surgical interventions into two types, local excision (LE) and radical excision (RE), to probe the relationship between surgery methods and survival outcomes. LE was defined as an intervention that resected or destroyed the primary mass locally, including damage surgery, partial resection, and palliative surgery [Site specific surgery (1973-1997) codes of 10-30,or Surgery of primary site (starting from 1998) codes of 10-32]. RE was defined as an intervention that resected the tumor radically, with a larger resection range than LE, including hemicolectomy, total colectomy, and proctocolectomy [Site specific surgery (1973-1997) codes of 40-50,70,or Surgery of primary site (starting from 1998) codes of40-70].

### Inclusion and exclusion criteria

There were 3 inclusion criteria in the current study: (1) Anatomic site of the primary tumor localized on the colon or the rectum (the International Classification of Disease for Oncology, Third Edition code, ICD-O-3: C180-189, C199 and C209); (2) Histological type limited to lymphoma (ICD-O-3 histology codes: 9590-9738); and (3) Malignant behavior (Behavior code ICD-O-3 code: 3). Mantle cell lymphoma (ICD-O-3 histology codes: 9673), diffuse large B-cell lymphoma (9680), Burkitt lymphoma (9687), follicular lymphoma (9690-9698) and marginal zone B-cell lymphoma (9699) were included in this analysis because these types constitute the vast majority of cases. The current study used 3 exclusion criteria: (1) Patients without histological confirmation (Diagnostic Confirmation codes: 2, 4, 5, 6, 7, 8, 9); (2) Patients with death certificate only (Type of reporting death code: 7); and (3) Patients with unclear information regarding surgery. Patients with multiple primary tumors without active follow-up or explicit survival results were also excluded from the survival study to improve the accuracy of survival analyses. Figure 1 shows the detailed screening procedure.

### Statistical analysis

Statistical analysis was performed using SPSS statistical software version 19.0 (SPSS Inc., IBM Corporation, Chicago, IL, USA) and R version 3.2.3 software (R Foundation for Statistical Computing, Vienna, Austria). Significant differences in patient demographics and tumor characteristics between the two groups were detected using the Pearson Chi-square test and the Wilcoxon rank sum test. We calculated the number of months from the date of diagnosis to the date of death to acquire OS. Univariate and multivariate models were established to evaluate correlations between various covariates and survival. The model-fitting methods were divided into three steps to ensure the quality of the regression analysis: variable selection, proportional hazards assumption verification and modeling, and goodness-of-fit assessment. Related references were consulted [11, 12, 27] to compare clinical experiences, age at diagnosis, gender, race, stage, tumor location, histological type, surgery, radiation and year of diagnosis, which were included as relevant covariates in the univariate analysis. The Kaplan-Meier method was performed to calculate the median and 10-year OS, and log-rank statistics were used to detect survival differences between the various covariates. We performed the Cox proportional hazard model to estimate the adjusted HRs between survival and covariates. Statistically significant covariates identified in univariate analyses (*P* < 0.1) were included in crude multivariate models, and their respective interactions were tested. Forward elimination was used to remove covariates with *P* >0.05 until all the remaining variables had *P* values < 0.05 in at least one stratum. The required proportional hazards assumption was tested and satisfied. The goodness-of-fit of the Cox model was evaluated using the Harrell concordance index.

Patients with missing data were excluded from the univariate and multivariate analyses. Two-sided *P*-values < 0.05 were considered statistically significant. All confidence intervals were set as 95% CI.

## SUPPLEMENTARY MATERIALS TABLES


