# Supplementary material for: The role of surgical intervention in primary colorectal lymphoma: A SEER population-based analysis
Source: Oncotarget. 2016 Sep 29;7(44):72263–75. doi: 10.18632/oncotarget.12344 (PMC5342160; doi:10.18632/oncotarget.12344)
Supplement: Supplementary file 1 [file oncotarget-07-72263-s001.pdf]

The role of surgical intervention in primary colorectal lymphoma: A SEER population-based analysis

Supplementary Material

Table S1.Characteristics of included patients with PCL for survival study.

Variable	Overall Patients	Included Patients [a]	P Value
**Total Patients (%)**	3342 (100%)	2198 (65.8%)	
**Age (SD)**			
Mean age	63.9±18.3	61.6±19.0	
**Gender (%)**			0.743 [b]
Male	2046(61.2%)	1336(60.8%)	
Female	1296(38.8%)	862(39.2%)	
**Race (%)**			0.572 [b]
White	2275(83.0%)	1790(81.4%)	
Black	189(5.7%)	131(6.0%)	
Asian	320(9.6%)	230(10.5%)	
Others	58(1.7%)	47(2.1%)	
**Stage (%)**			0.930 [b]
Stage IE	1396(41.8%)	902(41.0%)	
Stage IIE	767(23.0%)	505(23.0%)	
Stage IIIE	168(5.0%)	119(5.4%)	
Stage IVE	712(21.3%)	483(22.0%)	
Not applicable	114(3.4%)	77(3.5%)	
Unkown	185(5.5%)	112(5.1%)	
**Radiation (%)**	295(8.8%)	198(9.0%)	0.753[b]
**Surgery(%)**	2050(61.3%)	1360(61.9%)	0.691[b]
**Location (%)**			0.742 [b]
Cecum	1165(34.9%)	809(36.8%)	
Appendix	109(3.3%)	71(3.2%)	
Right-sided colon	616(18.4%)	392(17.8%)	

Left-sided colon	648(19.4%)	408(18.6%)	
Colon, NOS	358(10.7%)	221(10.1%)	
Rectum	446(13.3%)	297(13.5%)	
**Histology (%)**			0.106 [b]
NHL, large B-cell	1828(54.7%)	1215(55.3%)	
Marginal zone B-cell	618(18.5%)	373(17.0%)	
Burkitt lymphoma	233(7.0%)	192(8.7%)	
Follicular lymphoma	346(10.4%)	218(9.9%)	
Mantle cell lymphoma	317(9.5%)	200(9.1%)	
**Year of diagnosis (%)**			0.550 [b]
1973-1990	274(8.2%)	204(9.3%)	
1991-2000	753(22.5%)	486(22.1%)	
2001-2005	991(29.7%)	637(29.0%)	
2006-2011	1324(39.6%)	871(39.6%)	

Abbreviation: PCL, Primary Colorectal Lymphoma; SD, Standard Deviation; NOS, Not Otherwise Specified; NHL, Non-Hodgkin's Lymphoma.

[a] Included patients mean the eligible cases for survival study.

[b] Pearson Chi-square test.

Table S2. Survival Associated with Surgery Type.

**Surgery Type**	**Number(%)**	**Median OS (95%CI)**	**Number (OS<3m,%)**	**Mean age**	**Stage (%)**		**P value** [a]		
					**IE**	**IVE**	**LE**	**RE**	**NS**
LE	617(29.4)	144(106.0-182.0)	63(10.2)	60.2	54.7	17.4	-	0.003	<0.001
RE	647(30.8)	102(73.9-130.1)	102(15.8)	62.1	31.4	22.7	0.003	-	0.177
NS	838(39.9)	74(59.8-88.2)	122(14.6)	62.6	49.3	29.9	<0.001	0.177	-
P value		<0.001 [a]	0.010 [b]	0.231 [c]	<0.001 [b]	<0.001 [b]			

Abbreviation: OS, Overall Survival; CI, Confidence Interval; LE, Local Excision; RE, Radical Excision; NS No Surgery.

[a] the Log Rank test.

[b] Pearson Chi-square test.

[c] Wilcoxon rank sum test.
